# Viability, Enzymatic and Protein Profiles of *Pseudomonas aeruginosa* Biofilm and Planktonic Cells after Monomeric/Gemini Surfactant Treatment

**DOI:** 10.3390/molecules23061294

**Published:** 2018-05-28

**Authors:** Anna Koziróg, Anna Otlewska, Bogumił Brycki

**Affiliations:** 1 Institute of Fermentation Technology and Microbiology, Faculty of Biotechnology and Food Science, Lodz University of Technology, Wólczańska 171/173, 90-924 Lodz, Poland; anna.otlewska@p.lodz.pl; 2 Laboratory of Microbiocides Chemistry, Faculty of Chemistry, Adam Mickiewicz University in Poznan, Umultowska 89b, 61-614 Poznan, Poland; brycki@amu.edu.pl

**Keywords:** gemini surfactants (GS), antibiofilm activity, *Pseudomonas*, proteins profile, polipropylene

## Abstract

This study set out to investigate the biological activity of monomeric surfactants dodecyltrimethylammonium bromide (DTAB) and the next generation gemini surfactant hexamethylene-1,6-bis-(*N*,*N*-dimethyl-*N*-dodecylammonium bromide) (C6) against the environmental strain *Pseudomonas aeruginosa* PB_1. Minimal inhibitory concentrations (MIC) were determined using the dilution method. The viability of the planktonic cells and biofilm was assessed using the plate count method. Enzymatic profile was determined using the API-ZYM system. Proteins were extracted from the biofilm and planktonic cells and analysed using SDS-PAGE. The MIC of the gemini surfactants was 70 times lower than that of its monomeric analogue. After 4 h of treatment at MIC (0.0145 mM for C6 and 1.013 mM for DTAB), the number of viable planktonic cells was reduce by less than 3 logarithm units. At the concentration ≥MIC, a reduction in the number of viable cells was observed in mature biofilms (*p* < 0.05). Treatment for 4 h with gemini surfactant at 20 MIC caused complete biofilm eradication. At sub-MIC, the concentration of some enzymes reduced and their protein profiles changed. The results of this study show that due to its superior antibacterial activity, gemini compound **C6** can be applied as an effective microbiocide against *P. aeruginosa* in both planktonic and biofilm forms.

## 1. Introduction

Numerous microorganisms alternate between two models of growth: planktonic cells and biofilms. These organized communities of bacteria or microscopic fungi are able to grow on various surfaces, and are the main cause of many diseases. It is estimated that about 80% of all microbial infections are associated with biofilms [1]. Moreover, many bacteria can form biofilms on abiotic surfaces. These microorganisms are often responsible for biocorrosion and biofouling in diverse industry sectors. They can cause significant damage of equipment and increase maintenance costs [2].

One of the major biofilm−forming species is *P. aeruginosa*. An opportunistic pathogen, it is commonly found in soil and water (natural and artificial systems) and can contaminate food and drinking water [3]. Its motile rods are able to colonize various habitats, such as plant biomass used for renewable energy production. The strain *P. aeruginosa* PB_1 used in our study was found forming biofilm on the surface of a transporting belt carrying biomass from a heap to the furnace. *P. aeruginosa* is associated with a wide range of infections, especially among immunocompromised patients after surgical operations. It can from biofilms on medical equipment such as catheters, stents or different implants [4]. Its bacteria are responsible for about 57% of total nocosomial infections [5]. *P. aeruginosa* bacteria can cause urinary tract infections, peritonitis, burn wound infections or lung infections in cystic fibrosis sufferers.

Biofilm bacteria are physiologically different from planktonic forms [6,7]. The major feature of biofilms is the presence of extracellular polymeric substance (EPS) including not only polysaccharides, as was assumed in the past [8], but also proteins, lipids and extracellular DNA (eDNA). The self-produced biopolymer matrix binds the cells in the biofilm together. It provides both structural stability and protection [1,6,9,10]. Biofilms also display specific properties, including increased resistance to environmental changes, biocides or antibiotics [1,6,11,12,13]. Mature biofilms are the most difficult to eradicate, including because of the differences in the protein patterns between biofilms and planktonic cells [6]. The complex structure of the matrix in biofilms diminishes the activity of biocides, especially in terms of the diffusion process, which enables the biocidal particles to penetrate into deep layers [14]. New active biocides are therefore sought, able to inhibit biofilm formation or disassemble mature biofilm. Research is ongoing into biofilms, including their potential molecular mechanisms, signal transduction pathways and formation mechanisms on various surfaces [13,15]. Gemini surfactants, in particular cationic compounds with alkyl side chains, appear useful for degrading biofilms [16,17]. According to Paniak et al. [18], Jennings et al. [16] and Murguia et al. [19], compounds containing 12 carbon atoms per chain possess the best properties.

The aim of the presence study was to compare hexamethylene-1,6-bis-(*N*,*N*-dimethyl-*N*-dodecylammonium bromide) (C6) and the monomeric alkylammonioum salt *n*-dodecyltrimethylammonium bromide (DTAB), to determine their influence on the viability of planktonic cells and biofilm removal from polypropylene surfaces. The influence of the surfactants on enzymatic and protein profiles at sub-MIC was also investigated, to the best of our knowledge for the first time.

## 2. Results and Discussion

### 2.1. Minimal Inhibitory Concentration (MIC)

The effectiveness of gemini surfactant and monomeric analogues on *P. aeruginosa* PB_1 was examined by determination of minimal inhibitory concentration (MIC). For hexamethylene-1,6-bis-(*N*,*N*-dimethyl-*N*-dodecylammonium bromide) (C6), the MIC was 0.0145 mM. The monomeric surfactant *n*-dodecytrimethylammonium bromide inhibited *P. aeruginosa* growth at 70 times higher concentrations. The MIC value is 1.013 mM.

The antimicrobial activity of cationic GS on *P. aeruginosa* PAO_1 has also been examined by Paniak et al. [18]. The MIC value for pentamethylene-1,5-bis-(*N*,*N*-dimethyl-*N*-dodecylammonium bromide (C5), was 0.002 mM, while for heptamethylene-1,7-bis-(*N*,*N*-dimethyl-*N*-dodecylammonium bromide (C7) was 0.008 mM. Jennings et al. [16] obtained an MIC value of 0.004 mM for the same strain. These results are lower than those obtained for gemini surfactant C6 (12-6-12) at concentration of 0.0145 mM. It is worth noting that the present study used an environmental strain. Our previous research [20] demonstrated that environmental strains are less susceptible to biocidal compounds.

### 2.2. Biofilm Formation by P. aeruginosa

**Figure 1:**
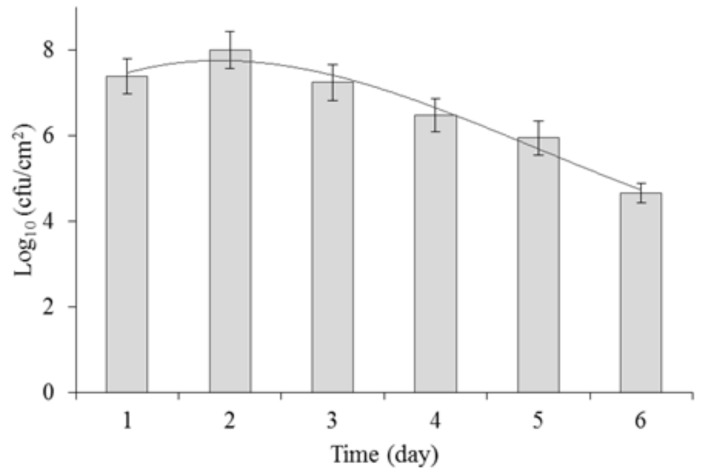
Growth of *P. aeruginosa* PB_1 biofilm on the surface of polypropylene in 6 days.

*P. aeruginosa* was cultivated for 6 days to determine its growth dynamics and biofilm formation on polypropylene. The number of cells adhered on the polypropylene surface was monitored every 24 h. The results are presented in Figure 1.

As reported by Garrett et al. [21], Meira et al. [22] and Rühs et al. [23], biofilm formation is affected by several factors, including the type of material, the cultivation medium, the pH, the temperature and oxygenation, which can also affect biofilm formation under the applied cultivation conditions. It is therefore important to determine the growth dynamics of a biofilm at an early stage of an experiment.

Previous studies on *P. aeruginosa* used 24 h [3,24], 48 h [9] or 72 h [25] biofilm. In our study, the most intensive growth of *P. aeruginosa* PB_1 was observed after 2 days of cultivation. The average number of microorganisms was 1.8 × 10^8^ cfu/cm^2^. After that, a gradual decrease in the number of viable cells was observed, up to 4.3 × 10^5^ cfu/cm^2^ after 6 days of incubation.

### 2.3. Effect of Surfactants on Pre-Formed Biofilm

**Figure 2:**
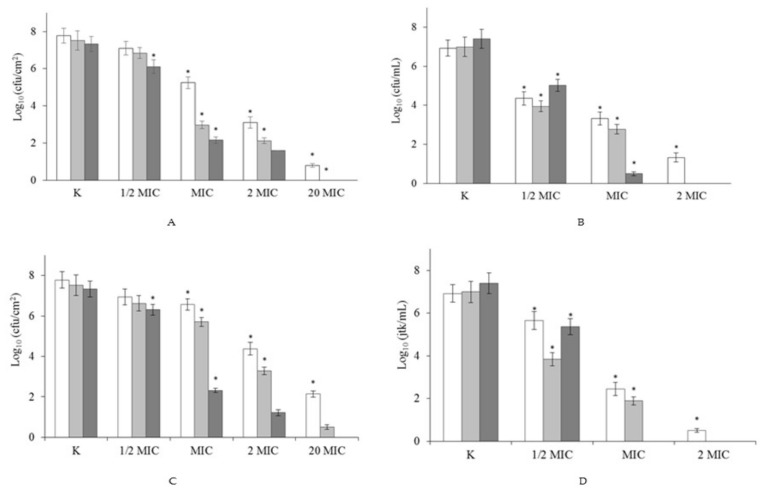
Effect of gemini C6 (**A**,**B**) and monomeric DTAB (**C**,**D**) surfactants on eradication of biofilm formed by *P. aeruginosa* (**A**,**C**) and planktonic cells (**B**,**D**). The results are presented after 1 h (white bar), 4 h (light grey bar) and 24 h (dark grey bar) treatment by biocides in the concentration ½ MIC, MIC, 2 MIC and 20 MIC, and compared to the control sample (K) without surfactants. * Reduced value of log_10_ differed significantly from the control without surfactant (K).

The next stage of our study investigated the ability of DTAB and C6 to eradicate 2-days biofilms formed on polypropylene. Four concentrations were tested: MIC (determined for planktonic cells), ½ MIC, 2 MIC and 20 MIC (Figure 2).

When the concentration of surfactants was ≥MIC, a reduction was observed in the number of bacteria forming biofilm (*p* < 0.05). Use of biocides at ½ MIC led to a statistically significant reduction in the number of cells after 24 h, of 1.2 log for C6 (Figure 2B) and of 1 log for DTAB (Figure 2D) in comparison to the control samples. Significant decreases in the number of viable biofilm-forming cells were detected after treatment with gemini surfactant or its monomeric analogue at MIC, 0.0145 mM and 1.013 mM, respectively. After 1 h of C6 activity, the number of bacteria rose to 5.25 log (Figure 2A), while after 1 h of exposure to DTAB it reached 6.6 log (Figure 2C). By comparison, after 24 h of treatment the number of bacteria fell to 2.16 and 2.32 log, for C6 and DTAB, respectively. Total biofilm eradication was achieved at 20 MIC after 4 h of treatment with C6, and after 24 h with DTAB. Even short-term exposure (1 h) to the highest concentration led to a considerable reduction of 7 log for C6, and of 5.64 log for DTAB. A greater reduction in the number of biofilm-forming cells was observed following treatment with gemini surfactant. This is in agreement with our previous research [17], which proved that DTAB and C6 are highly effective at removing *Asaia lannensis* biofilm.

It is worth remembering that bacterial biofilms are less sensitive to biocides than planktonic cells. Moreover, the eradication process is often very difficult to perform. This may be due to the extracellular matrix in biofilms, or to changes in their metabolic activity [3,6,9,26,27]. Little is known about the biofilm-eradicating properties of quaternary ammonium salts (QAS). However, according to Jennings et al. [16] and Obłąk et al. [28], gemini surfactants can effectively remove biofilms. Jennings et al. [16] applied gemini surfactant in research on pentamethylene-1,5-bis-(*N*,*N*-dimethyl-*N*-dodecylammonium bromide) C5, which after 24 h of treatment at a concentration of 0.075 mM inhibited the growth of 24 h biofilms of *S. aureus* and *E. faecalis*. By comparison, Obłąk et al. [28] found that TMEG-12 Br at a concentration 0.12 mM was highly effective for the eradication of *P. aeruginosa* PAO1 biofilm. In the present study, gemini surfactant C6 had the same effect against the environmental *P. aeruginosa* strain PB_1 at a concentration of 0.29 mM. In the next stage of our research, we applied compounds at identical concentrations (sub-MIC determined for planktonic cells), to compare the changes in the protein and enzymatic profiles of planktonic cells and biofilms.

### 2.4. Influence of Surfactants on Enzymatic Profiles of Biofilms and Planktonic Cells

Both clinical and environmental *P. aeruginosa* strains are able to produce large array of extracellular enzymes. Studies on biofilms produced by *P. aeruginosa* PB_1 on polypropylene revealed the activity of seven enzymes, i.e., alkaline phosphatase, esterase (C4), esterase lipase (C8), lipase (C14), leucine arylamidase, acid phosphatase and napthol-AS-BI-phosphohydrolase. The same results were obtained when planktonic cells were examined without biocides. Interestingly, when the surfactants were added to planktonic cultures, the expression of several enzymes was not suppressed. This was the case with esterase lipase (4), except when treated with DTAB at ¼ MIC, under which condition the activity of this enzyme was higher; with leucine arylamidase (6) when DTAB was added at ¼ MIC and with acid phosphatase (11) after 24 h of treatment with gemini surfactant at ¼ and ½ MIC. Esterase lipase (4) was the only enzyme which showed higher activity in a sample treated with DTAB at ¼ MIC after 4 and 24 h. Several enzymes were not detected in the planktonic cells of *P. aeruginosa*: alkaline phosphatase (2) (surfactant C6 at both concentrations and DTAB at ½ MIC), lipase (5) (C6 at ½ MIC and DTAB at ½ MIC only after 24 h), acid phosphatase (11) (DTAB at ½ MIC) and napthol-AS-BI-phosphohydrolase (12) (C6 at both concentration). The concentration of the remaining enzymes decreased.

**Figure 3:**
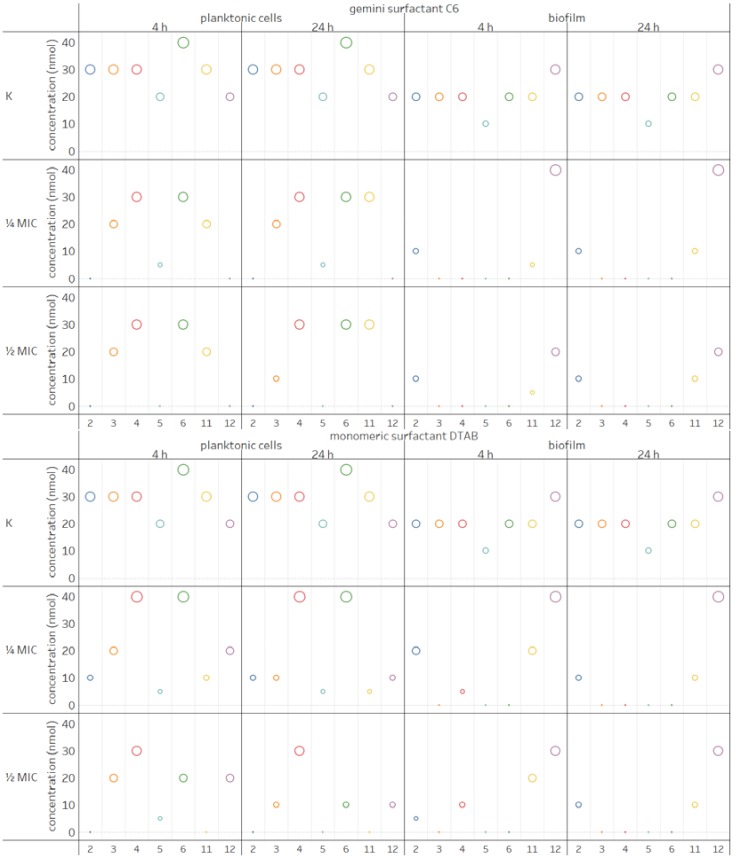
Effect of gemini C6 and monomeric DTAB surfactants at ¼ and ½ MIC concentration on enzymatic profile of *P. aeruginosa* in biofilm and planktonic cells (the numbers 2–12 plotted on the horizontal axis refer to the enzyme number in the API ZYM test: 2—alkaline phosphatase, 3—esterase (C4), 4—esterase lipase (C8), 5—lipase (C14), 6—leucine arylamidase, 11—acid phosphatase and 12—napthol-AS-BI-phosphohydrolase, respectively).

Less diversity was observed in the enzymatic profiles of biofilm formed by *P. aeruginosa* after treatment with gemini surfactant and its monomeric analogue, compared to the sample without biocides. After treatment with surfactants, we did not detect the activity of esterase (3), esterase lipase (4) (excluding in the case of treatment with DTAB at ¼ and ½ MIC after 4 h), lipase (5) or leucine arylamidase (6) in any sample. On the other hand, the concentration of napthol-AS-BI-phosphohydrolase (12) was higher both for C6 and DTAB at ¼ MIC, while the activity of acid phosphatase (11) decreased after use of gemini surfactant (24 h and 48 h) and the monomeric compound (24 h) at both concentrations. In summary, the enzymatic profiles of *P. aeruginosa* presented in Figure 3 show that both C6 and DTAB had a weaker impact on planktonic cells than biofilms. In addition, 4-h treatment of biofilms with gemini surfactant resulted in greater changes in the enzymatic profiles, in comparison to its monomeric analogue.

Extracellular enzymes are important virulence factors. They are responsible for the survival of cells in unfavourable conditions [4,5] and have an impact on the composition and physic-chemical properties of extracellular polymeric substances (EPS) during subsequent stages of biofilm formation. This is particularly the case with lipolytic and proteolytic enzymes [3]. *P. aeruginosa* produces two lipases [4]. These enzymes can bind to alginate, which leads to accumulation in *P. aeruginosa* mucoid strains [26]. They also play an important role in pathogenesis. Combined with hemolytic phospholipase C, they can degrade lung surfactant lipid [4]. In contrast, alkaline phosphatase (*P. aeruginosa* PB_1 contained this enzyme) was found in membranes vesicles (MVs). They are spherical structures which are secreted into the environment by some Gram-negative bacteria. Although, alkaline phosphatase is not directly associated with pathogenesis, it is a periplasmatic marker in *P. aeruginosa* and other Gram-negative bacteria [29]. Therefore, decrease in the concentration of extracellular enzymes as a result of treatment with monomeric and gemini surfactants may contribute to the reduction of pathogenicity of *P. aeruginosa*.

### 2.5. Changes of Proteins Profile in Biofilm and Planktonic Cells after Surfactant Treatment

Whole cell protein analysis of *P. aeruginosa* PB_1 revealed 33 protein fractions with molecular weights ranging from 20 to 289 kDA for planktonic cells and 19–21 protein fractions from 21 to 272 kDa for biofilm.

When the number of protein fractions in planktonic cells after treatment with monomeric or gemini surfactants were compared with the number in untreated planktonic cells, no significant differences were observed. The similarity between the profiles of these samples and those determined by calculating the Dice index was <95% (Table 1). The Dice coefficient was equally high for biofilms treated with DTAB, while for hexamethylene-1,6-bis-(*N*,*N*-dimethyl-*N*-dodecylammonium bromide) C6 it decreased to 50% at ¼ MIC and 17.4% at ½ MIC (Table 1). Significant changes in protein profiles were thus observed exclusively for biofilms treated with C6 compound at sub-MIC.

**Figure 4:**
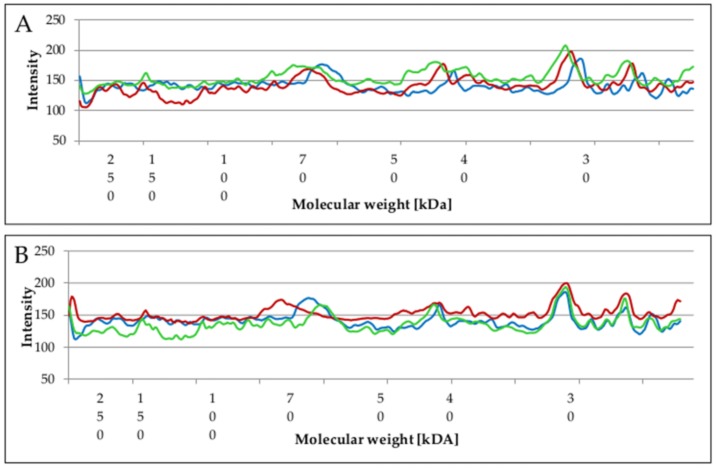

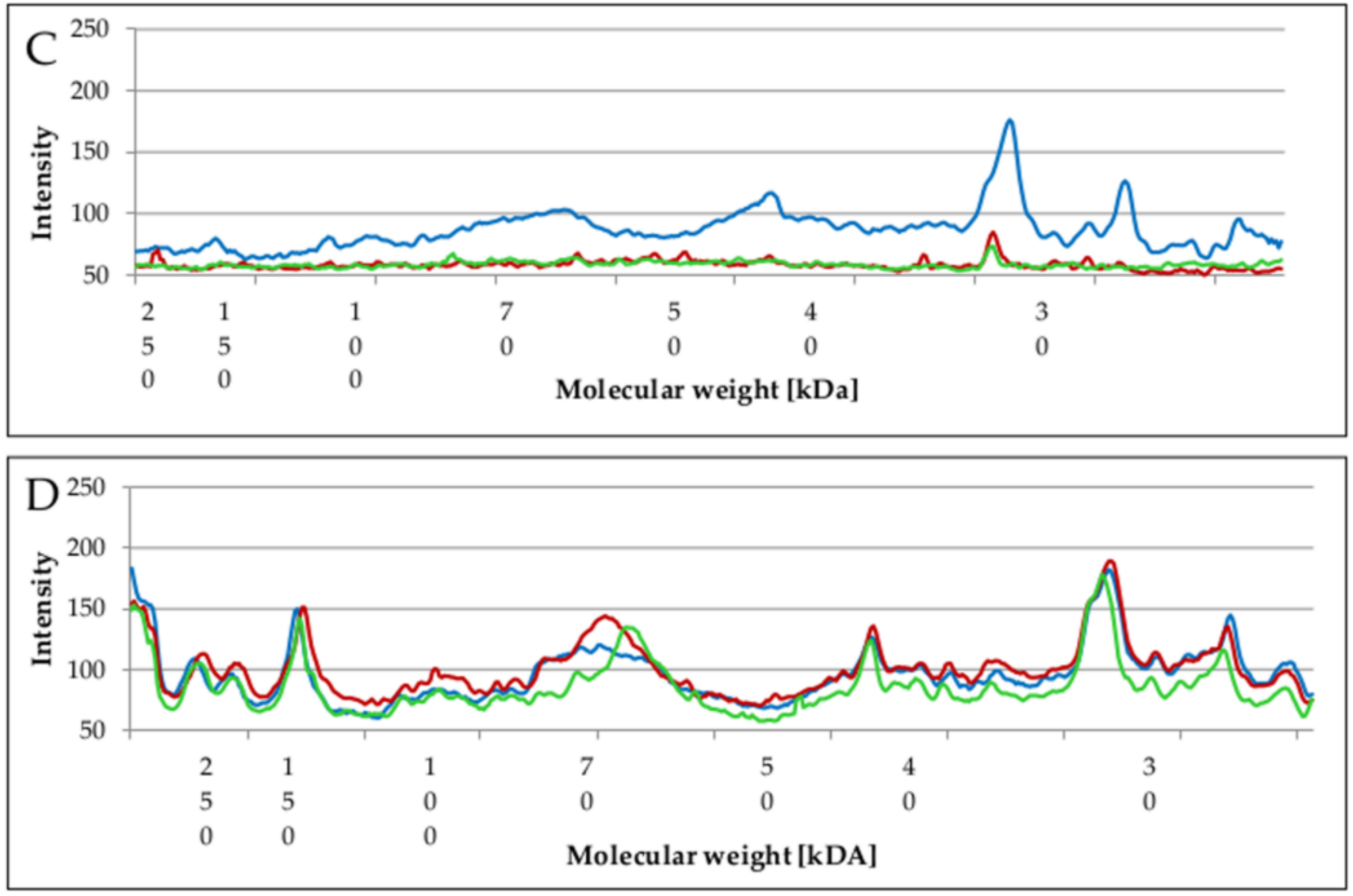
Changes in pixel intensities of protein profiles in *P. aeruginosa* PB_1 planktonic cells (**A**,**B**) and biofilm (**C**,**D**) after gemini surfactant C6 (**A**,**C**) and monomeric surfactant DTAB (**B**,**D**) treatment at ¼ MIC concentration (red line), ½ MIC concentration (green line) in comparison to the sample without biocide (blue line).

Considerable differences in protein profiles were visible when the pixel intensities of the protein bands were compared. The intensities of individual bands were determined using SDS-PAGE. Figure 4 shows the relationship between proteins molecular weight and pixel intensities.

A comparative analysis of the protein profiles revealed higher pattern intensity (above 120) in planktonic cells than in biofilm. After treatment of planktonic cells with C6 compound, the intensity of protein bands increased. This was especially the case in the range of 25–50 kDa compared to the untreated sample (Figure 4A).

By comparison, the highest intensity in the entire range of molecular weight was observed at ¼ MIC (Figure 4B) after treatment with DTAB. Protein bands intensity was highest when the biofilm was treated with DTAB at ¼ MIC. However, there were slight differences between the samples, depending on the MICs (¼ MIC, ½ MIC or K).

**Figure 5:**
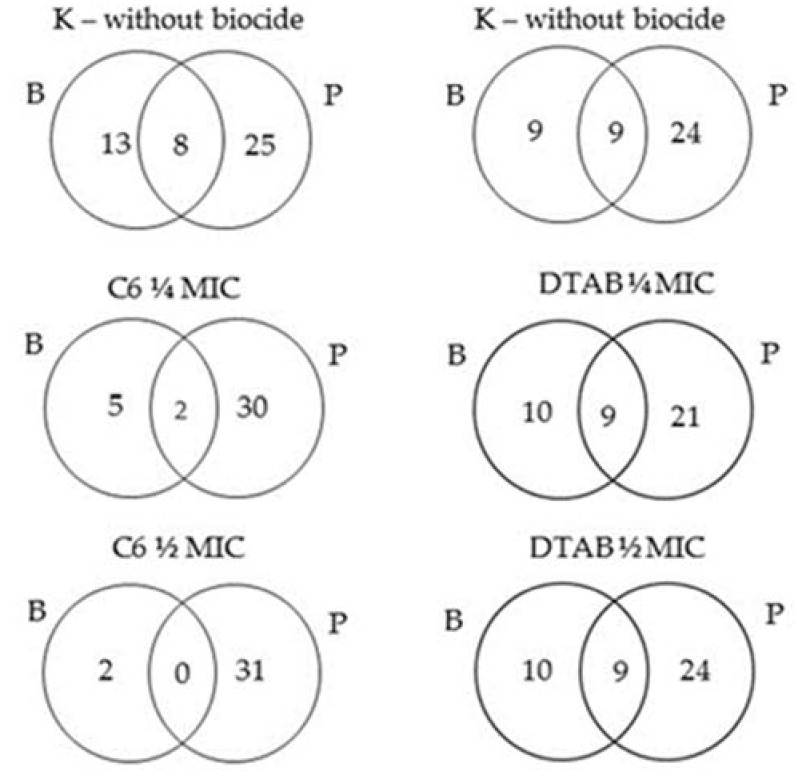
Venn diagram showing the numbers of protein bands in biofilm (B) and planktonic cells (P) o *P. aeruginosa* PB_1 after 24-h treatment with gemini C6 and monomeric DTAB surfactants.

Increased proteins profile intensity may be a defensive reaction triggered when cells are treated with compounds at sub-MIC, which do not entirely inhibit growth. In particular, those proteins that are bound to an extracellular matrix form an integral system, which contributes to stress resistance and the stability of the biofilm [27]. Much lower protein pattern intensity was observed for C6 compound in comparison to the untreated sample (Figure 4C). This was in agreement with the Dice coefficient. A similar relationship was noted by Shafiei et al. [30], who treated biofilm formed by *P. aeruginosa* strain PAO1 with *n*-butanolic *C. coum* extract and ciprofloxacin. Dissimilarities were also observed in the number of isolated proteins derived from *P. aeruginosa* PB_1 (Figure 5).

The number of proteins was higher in planktonic cells regardless of the sample. After treatment with monomeric surfactant DTAB the differences were slight when compared to the samples without biocide. Biofilm and planktonic cells had 9 identical protein bands. On the other hand, when the concentration of gemini surfactants increased, the number of isolated proteins from biofilm decreased (Figure 5). When C6 compound was used at ½ MIC concentration, no identical proteins were found both in planktonic cells and biofilm.

According to Sauer et al. [6], changes in protein profiles occur throughout the development of biofilms, starting with reversible attachment up to final dispersion. These protein patterns varied from those observed in planktonic forms. The results of our study clearly demonstrate disproportions between the proteins in the biofilms and planktonic cells. Both Sauer et al. [6] and Shafiei et al. [30] extracted larger number of proteins from biofilm. However, Toyofuku et al. [9] and Couto et al. [31] found lower numbers of proteins in outer membrane vesicles (OMV) in biofilms of *P. aerugionosa* PAO1 compared with planktonic cells. Such differences may be explained by numerous factors. Firstly, in our preliminary study regarding the changes in protein profiles of *P. aeruginosa* PB_1 treated with monomeric and gemini surfactants, we applied SDS-PAGE electrophoresis. Shafiei et al. [30], Couto et al. [31] and Toyofuku et al. [9] used two-dimensional electrophoresis. Secondly, according to our previous research [17] different cultivation strategies and environmental parameters (such as pH, medium composition or time) may influence biofilm formation. In the present study, we investigated 48 h biofilm formed on polypropylene and performed experiments with shaking. Finally, as demonstrated by Toyofuku et al. [9], differences in the protein content of planktonic cells and biofilm may be related to the various physiological states of cells. Antimicrobial agents themselves may also have an impact.

Gemini surfactant C6 caused greater changes to the enzymatic and protein profiles than its monomeric analogue. The used of compounds at sub-MIC enabled us to observe the changes in the biofilms and planktonic cells. Antimicrobial agents at sub-MIC often trigger defence reactions, which may explain the increased synthesis of several components in the biofilm matrix or cell wall in the planktonic structure. It worth noting that compounds at such concentration contribute to growth inhibition. Cells which remain in biofilm may adapt to the unfavourable conditions and become more resistant [32]. According to Aka et al. [33], clinical isolates of *P. aeruginosa* in the presence of chlorhexidine at sub-MIC are able to form biofilm even when antibiotics are added. This highlights the great importance of respecting the recommended concentrations of both antibiotics or biocides.

Quaternary ammonium salts have been known for over a hundred of years and are one of the most commonly used compounds for disinfection. As a result, increasing numbers if microorganisms are becoming less susceptible to QAS. Resistant forms of microorganisms identify monocationic and conformationally-rigid structures in monomeric surfactants. These positively-charged compounds ionically interact with negatively charged components in the biofilm matrix, reducing penetration. The same effect occurs when it comes to positively charged antibiotics such as tobramycin [32]. However, next-generation QAS such as gemini surfactants, owing to their higher charge and conformational flexibility, do not activate resistance [2]. One of the possible mechanisms by which polycationic compounds can eradicate biofilms is through electrostatic interactions, which interfering with their functioning. The cells in the biofilm matrix are then killed by cell lysis [34]. The compounds presented in our study are a relatively new group, but their effectiveness depends on appropriate application.

## 3. Materials and Methods

### 3.1. Microorganisms and Technical Materials

Experiments were performed with *Pseudomonas aerugionsa* PB_1, isolated from plant biomass in Poland. The strain was identified by molecular methods based on 16S rRNA gene sequencing. The nucleotide sequence of the 16S rRNA gene were deposited in the NCBI GenBank database as MF777034. The biological material was stored in Tryptic Soy Agar (TSA, Merck, Darmstad, Germany) at 4 °C. Rectangular pieces of polypropylene (PP) 70 × 25 mm (Packor Packaging, Skierniewice, Poland) were used in the adhesion test. The test material was certified by the Polish National Institute of Public Health [35].

### 3.2. Antimicrobial Agents

The antimicrobial agents used were the dimeric alkylammonium salt hexamethylene-1,6-bis-(*N*,*N*-dimethyl-*N*-dodecylammonium bromide) (C6) and the monomeric alkylammonioum salt *n*-dodecyltrimethylammonium bromide (DTAB). The gemini surfactant (C6) was synthesized by the reaction described by Koziróg et al. [17]. Dodecyltrimethylammonium bromide is commercially available (Aldrich, Munich, Germany).

### 3.3. Minimal Inhibitory Concentration

The MIC values for the bacteria were determined using the tube standard two-fold dilution method, as described by European Normative EN 1276 [36]. Incubation was conducted on a rotary shaker (100 rpm) for 24 h at 37 °C in Tryptic Soy Broth medium (TSB; Merck). The bacterial culture was centrifuged (8000 rpm for 5 min) and supernatant was separated. The bacterial biomass was suspended in tryptophan sodium chloride solution. The cell level was 1.5 × 10^8^ cfu/mL. In the next step, to each tube containing 1 mL of TSB was added 1 mL of a solution containing C6 compounds at a concentration of 7.454 mM (0.5%) or DTAB at a concentration of 16.216 mM (0.5%). Serial dilutions of the tested compounds with 1 mL of medium were mixed with 1 mL of the cell suspension. Finally, the samples were incubated at 37 °C for 24 h. Bacterial suspensions in culture media without the tested biocides were used as control samples (1 mL medium + 1 mL bacterial suspension 1.5 × 10^8^ cfu/mL). The MICs were defined as the lowest concentrations of the compounds in which there was no visible growth of *P. aeruginosa*.

### 3.4. Biofilm Formation

Biofilm was prepared in flasks containing 18 mL TSB, polypropylene rectangle and 2 mL of bacterial suspension (10^7^ cfu/mL). The samples were incubated at 25 °C on a laboratory shaker (100 rpm) for 1–6 days. The PP rectangles were then removed from the culture medium, rinsed with sterile distilled water and swabbed using sterile swabs. The tested surface covered 6.25 cm^2^. The swabs were then transferred to tubes containing 1.5 mL of saline (0.85% *w*/*v*). The number of cells in the biofilm was determined using the plate count method. The results were expressed in cfu per square centimetre.

### 3.5. Effect of Surfactants on Pre-Formed Biofilm and Planktonic Cells

In further research, 48 h biofilm was used according to the methodology mentioned above. After 2 days, the rectangular pieces of PP were removed from the culture medium and rinsed with sterile distilled water. In the next step, the PP pieces were transferred to flasks containing 18 mL PBS buffer and 2 mL monomeric or dimeric surfactant at ½ MIC, MIC, 2 MIC, or 20 MIC. A control sample (K) was transferred to PBS buffer without antimicrobial agents. After 1, 4 and 24 h, the PP rectangles were removed from the culture media and placed in 20 mL of saline with a mixture of neutralizers (5% Tween 80, 2% lecithin and 0.5% sodium thiosulfate) for 10 min [37]. The PP material was then rinsed with sterile distilled water and swabbed using sterile swabs (testing surface 6.25 cm^2^). Subsequently, the swabs were transferred to the tubes containing 1.5 mL of saline (0.85% *w*/*v*). The number of cells in the biofilm was determined using the plate count method. The results were expressed in cfu per square centimetre.

To determine the influence of the surfactants on planktonic cells, a liquid culture *P. aeruginosa* PB_1 was prepared. To 16 mL of TSB liquid medium were added 2 mL monomeric DTAB or gemini C6 surfactant, at ½ MIC, MIC or 2 MIC. Each test flask was inoculated with 2 mL of bacterial suspension with a bacterial cell level of approximately 10^7^ cfu/mL. Subsequently the samples were incubated at 37 °C on a laboratory shaker (100 rpm). After 1, 4 and 24 h, 1 mL of each mixture was transferred to 9 mL of 0.85% (*w*/*v*) saline with a mixture of neutralizers (5% Tween 80, 2% lecithin and 0.5% sodium thiosulfate) for 10 min [37]. The number of viable cells was determined on TSA agar medium (Merck) using the conventional plate count method. After incubation at 37 °C for 24 h, the colonies of *P. aeruginosa* were counted.

### 3.6. Enzymatic Profile in Biofilm and Planktonic Cells after Surfactant Treatments

To determine enzymatic profiles of *P. aeruginosa* PB_1 was used the API-ZYM test (bioMérieux, Marcy l’Etoile, France) The activities of 19 enzymes were measured: alkaline phosphatase, esterase (C4), esterase lipase (C8), lipase (C14), leucine arylamidase, valine arylamidase, cystine arylamidase, trypsin,α-chymotrypsin, acid phosphatase, naphthol-AS-BI-phosphohydrolase, α-galactosidase, β-galactosidase, β-glucuronidase, α-glucosidase, β-glucosidase, *N*-acetyl-β-glucosaminidase, α-mannosidase, and α-fucosidase. The biofilm and planktonic cells were prepared according to the methodology described above. Two-day biofilms and planktonic cells were treated with compounds at concentrations of ¼ and ½ MIC for 4 and 24 h. The density of the cell suspension used for the API-ZYM tests was 10^8^ cfu/mL.

### 3.7. Preparation of Protein Extracts

The same samples were used for protein analysis as in enzyme analysis. For whole cell protein extraction, 1.5 mL of biofilm or planktonic cells of *P. aeruginosa* (density 10^8^ cfu/mL) were harvested by centrifugation (12,000 rpm, 5 min) and washed twice with distilled water. Pellets were re-suspended in extraction buffer (0.1 M phenylmethylsulfonyl fluoride; 1.0 M ethylenediaminetetraacetic acid; 10% 2-mercaptoethanol), incubated for 15 min and centrifuged (12,000 rpm, 10 min). The pellets were mixed with loading buffer (0.06 M Tris-HCl pH 6.8; 10% glycerol; 2% sodium dodecyl sulphate; 5% 2-mercaptoethanol; 0.025% bromophenol blue) and boiled at 100 °C for 5 min. The protein concentration was determined using a NanoPhotometer™ Pearl UV-Vis spectrophotometer (Implen GmbH, München, Germany), according to the manufacturer’s protocol.

### 3.8. Protein Profile Analysis by SDS-PAGE

Whole cell proteins were analysed by sodium dodecyl sulphate polyacrylamide gel electrophoresis (SDS-PAGE) according to the method described by Laemmli [38]. Electrophoresis were performed in 5% stacking gel and 10% separating gel in Tris-Glycine-SDS buffer (25 mM Tris-HCl pH 8.6; 192 mM glycine; 0.1% sodium dodecyl sulphate) with using PROTEAN System (Bio Rad Laboratories Inc., Hercules, CA, USA) with a constant voltage of 20 V/cm. The gels were stained with Coomassie Brillant Blue R-250 (0.25% Coomassie Brillant Blue R-250; 10% acetic acid; 45% methanol) [39]. The molecular weight of the proteins and the pixel intensity of the protein bands were analysed with the SigmaGel software (Jandel Scientific, San Rafael, CA, USA). PageRuler Protein Ladder (Thermo Fisher Scientific, Waltham, MA, USA) was used as a size standard for protein electrophoresis. The average similarities between protein profiles were calculated by the Dice coefficient [40]:

S (%) = 2X × 100/(a + b)

where a is the total number of bands for the control, b is the total number of bands for the isolate, and X is the total number of similar bands in both compared lanes.

### 3.9. Statistical Analysis

Three independent experiments were performed. The mean results were calculated, with their standard deviations. Statistical differences in the data were compared using a one-way repeated measures analysis of variance (ANOVA; OriginPro 9.2.214, OriginLab Corp., Northampton, MA, USA). Statistical significance was set at 5% (*p* < 0.05).

## 4. Conclusions

Hexamethylene-1,6-bis-(*N*,*N*-dimethyl-*N*-dodecylammonium bromide), a member of the gemini surfactants group, exhibit high antimicrobial efficacy against *P. aeruginosa* PB_1. In this study, the compound reduced the number of viable bacterial cells in the planktonic state even at the very low concentrations of 0.0145 mM, while at 0.29 mM it proved to be effective at eradicating biofilm. Moreover, enzyme concentration in both planktonic cells and biofilms were reduced at sub-MIC, especially in the case of lipases. At sub-MIC, the protein profiles were also changed. The results of this study suggested that gemini surfactant could be a useful active compound for use in biocides against not only planktonic cells, but also biofilms on various abiotic surfaces.

## Figures and Tables

**Table 1:** Similarity of proteins profiles of *P. aeruginosa* PB_1 after monomeric DATB and gemini surfactant C6 treatment.

Biocide	Concentration	Planktonic Cells	Biofilm
K (control without biocide)		100	100
C6	¼ MIC 4 h	96.8	100
	¼ MIC 24 h	98.5	50
	½ MIC 4 h	98.5	100
	½ MIC 24 h	96.8	17.4
DTAB	¼ MIC 4 h	95.2	84.8
	¼ MIC 24 h	95.2	97.3
	½ MIC 4 h	96.9	97.3
	½ MIC 24 h	100	97.3
